# Favorable long term effects of intensified immunosuppression combined with therapeutic plasma exchange in patients with early-onset progressive systemic sclerosis-related interstitial lung disease^☆^

**DOI:** 10.1016/j.jtauto.2022.100174

**Published:** 2022-11-21

**Authors:** J. Potjewijd, R. Tobal, D. Silvertand, H.A. Gietema, J.G.M.C. Damoiseaux, P. van Paassen

**Affiliations:** a Department of Internal Medicine, Division Nephrology and Clinical Immunology, Maastricht University Medical Center, Maastricht, the Netherlands; b Department of Radiology and Nuclear Medicine, Maastricht University Medical Center, Maastricht, the Netherlands; c Diagnostic Laboratory, Maastricht University Medical Center, Maastricht, the Netherlands

**Keywords:** SSc, Systemic Sclerosis, ILD, Interstitial lung disease, PAH, Pulmonary Arterial Hypertension, TPE, Therapeutic Plasma Exchange, EOc, Estimated Optimal care, EFS, Event Free Survival, SMR, Standardized Mortality rate

## Abstract

**Objective:**

Systemic sclerosis (SSc) related mortality and morbidity remains high. Immunosuppressive therapy is considered most effective when immune activity and inflammation but not fibrosis still dominates the disease process. This study evaluated long-term intensified immunosuppression combined with therapeutic plasma exchange (TPE) in early-onset progressive SSc-related interstitial lung disease (ILD).

**Methods:**

The study cohort consisted of 161 SSc patients, with a median follow-up time of 8.9 years. The standardized mortality rate (SMR) and overall survival was calculated in patients with and without cardiopulmonary involvement. We used a standardized, pragmatic, non-randomized approach to treat 24 consecutive early progressive SSc-ILD patients with intensified immunosuppressive therapy, including plasma exchange. Outcome measurements were event-free survival (EFS), pulmonary function and safety profile. The outcome was compared with the analyzed data from the other SSc-ILD patients, who did not fulfill the inclusion criteria, and instead were treated with estimated optimal care (EOc).

**Results:**

The age-adjusted SMR of all 161 SSc patients was 3.0 (CI95%; 0.32–5.68). EFS at 10 years was 49.9% in the intensified treatment group and 43.3% in the EOc group (p = 0.106). Improvement of the percentage of predicted forced vital capacity (%pFVC) and percentage of predicted diffusing capacity for carbon monoxide (%pDLco) in the intensified treatment group was +10.1% respectively +3.6%, compared to a decrease of respectively 10.8% and 7% in the EOc (p < 0.001 resp. p = 0.019). Safety analysis showed 1 death (female patient, over 75 years of age), due to pneumosepsis, in the intensified treatment group.

**Conclusion:**

Intensified and long-lasting immunosuppression combined with TPE is safe in early severe systemic sclerosis and is associated with improved EFS and pulmonary function as compared to the outcome in the variable but EOc group. Our findings warrant larger studies for confirmation.

## 1 Introduction

Systemic sclerosis (SSc) is characterized by fibrosis of the skin and internal organs, small-vessel vasculopathy and immune dysregulation. Prevalence is low but SSc-related morbidity and mortality is high and a great burden to the patient and their relatives [1]. Interstitial lung disease (ILD) and pulmonary arterial hypertension (PAH) are the main causes of death in SSc [2,3]. Treatment encompasses four major different domains: general conservative measures such as statins and vasodilators, the use of different classes of PAH medication, immunosuppression, including stem cell transplantation, and more recently, antifibrotic therapy.

At least three randomized controlled studies demonstrated superior efficacy of autologous hematopoietic stem cell transplantation (HSCT) in selected SSc patients [[4], [5], [6]]. Treatment in high-risk patients with rapidly progressive SSc improved survival and prevented major organ failure compared to one year cyclophosphamide pulse therapy, however at the cost of high treatment-related mortality up to 10% [7]. HSCT is only available in highly specialized centers and patient selection is vital. Moreover, the comparator immunosuppression may not have been optimized in previous studies. Thus, alternative treatment regimens particularly in early progressive and severe disease are needed in order to prevent the long-term fibrotic sequelae and reduce SSc-related morbidity and mortality. In 2004 we initiated a pragmatic, non-randomized prospective protocol using a standardized diagnostic approach followed by intensified immunosuppressive therapy in addition to therapeutic plasma exchange (TPE) in all consecutive SSc-ILD patients fulfilling our strict inclusion criteria.

We now first report the calculated standardized mortality rate (SMR) and overall survival in the entire SSc cohort including patients with and without cardiopulmonary involvement, who visited our university hospital referral center between 2004 and 2017. Second, we show the long-term follow-up event-free survival (EFS), the changes in pulmonary function and safety profile of the early progressive SSc-ILD cohort. These patients were treated according to our intensified immunosuppression protocol. We compared the outcome with analyzed data from the SSc-ILD patients who did not fulfill the strict inclusion criteria.

## 2 Patients and methods

### Ethical statement

The protocol was initially designed to prospectively evaluate in a standardized, non-randomized fashion, the clinical effects of long-term immunosuppressive therapy in combination with short-term cycles of TPE in patients with early progressive systemic sclerosis and pulmonary involvement. Patients were informed and consented that no standard therapy was available at that time. Finally, we retrospectively evaluated all data for efficacy and safety. Ethical approval was obtained from the Medical Research Ethics Committee of Maastricht University Medical Center (MUMC+) (MEC- 2021–3010) and a waiver for informed consent was received.

### 2.1 Study population

Adults with systemic sclerosis were included if they visited the hospital between January 1, 2004 and July 1, 2017, and retrospectively met the 2013 ACR/EULAR criteria for SSc [7]. During follow-up until September 1, 2019, development of ILD and/or PAH was monitored. SSc-ILD was diagnosed in the presence of ground glass opacification or fibrosis on high resolution computed tomography (HRCT) and/or evidence of active alveolitis on 99 m Technetium-diethylenetriamine pentaacetate (99mTc DTPA) scan. PAH was defined as elevated mean pulmonary arterial pressure (mPAP) ≥25 mmHg in rest and supine position measured by right heart catherization of the pre-capillary type (pulmonary capillary wedge pressure (PCPW) ≤ 15 mmHg) and a pulmonary vascular resistance (PVR) > 3 wood units (WU) [8]. Patients were defined as SSc-PAH in this study when pre-capillary PH was present in the absence of significant ILD, meaning less than 10% lung fibrosis evaluated on HRCT. Clinical assessments including pulmonary function tests, HRCT and cardiac ultrasound were at least repeated annually during follow-up.

Inclusion criteria for the intensified immunosuppressive therapy group were a disease duration <3 years (defined as first non-Raynaud phenomenon (RP)), age above 18 years, and pulmonary involvement. Pulmonary involvement was defined as percentage of predicted diffusing capacity for carbon monoxide corrected for hemoglobin and alveolar volume (%pDLco)≤85%, and/or percentage of predicted forced vital capacity (%pFVC) ≤85% and evidence of ILD on HRCT; or %pDLco and/or %pFVC >85%, but with a progressive course of pulmonary disease defined as relative decline of >10% in %pFVC or >15% decline in %pDLco within 6 months ór pDLco and/or pFVC >85% and evidence of active alveolitis on 99mTcDTPA scan. Only immunosuppressive and antifibrotic treatment naïve patients were included. A low dose of prednisone <10 mg was allowed, as was the presence of PAH.

**Fig. 1:**
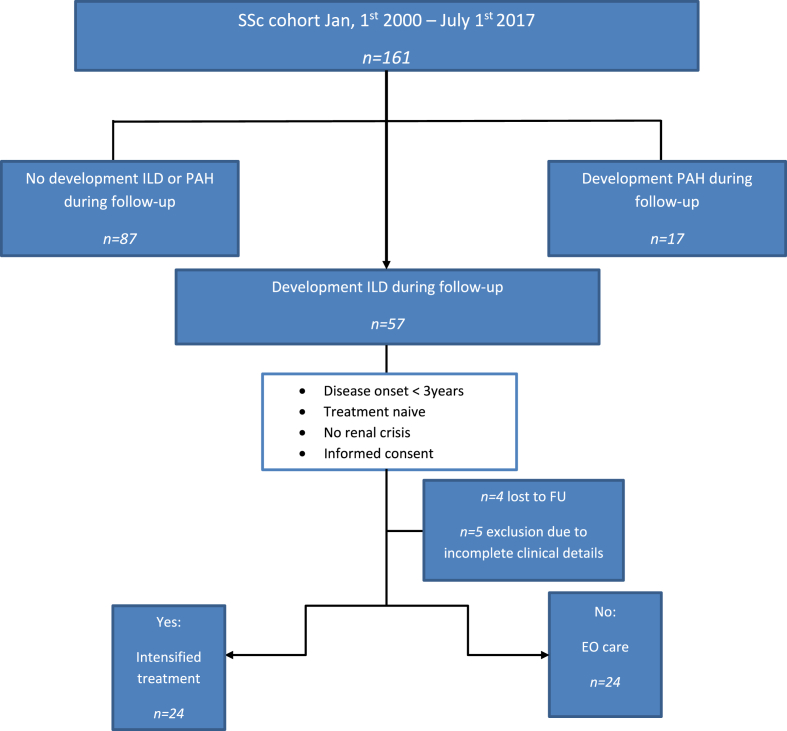
Procedure of patient enrollment. ILD: interstitial lung disease; EOc: estimated optimal care. PAH: pulmonary arterial hypertension, patients were defined as SSc-PAH in this study when pre-capillary PH was present in the absence of significant ILD, meaning less than 10% lung fibrosis evaluated on HRCT thorax.

In order to compare outcome measures of the intensified treatment group we selected a control group by including all patients from the complete SSc cohort, who fit the same definition of pulmonary involvement as the intensified treatment group. However, these patients were either not treatment naïve, suffered from earlier renal crisis, refused to give informed consent for TPE, or had a disease duration >3 years. They were treated with what was considered estimated optimal care (EOc) by their treating physician. Procedure of enrollment is shown in Fig. 1. Patients were considered lost to follow-up if the vital status was not confirmed. For these patients, vital status and date of last visit was noted.

### 2.2 Data collection

Data were retrospectively retrieved from medical records. Scleroderma renal crisis was defined as new onset malignant hypertension and oligo-/anuric renal failure. Gastrointestinal tract involvement included reflux, dysmotility, diarrhea, or signs of malabsorption. Cardiovascular disease was defined as ischemic heart failure or cerebral infarction. Antinuclear antibody (ANA) was tested by indirect immunofluorescence assay (IIFA) on HEp-2 cells, followed by specific extractable nuclear antigens (ENA) test and SSc blot of Euroimmun.

HRCTs were scored by an experienced radiologist, according to the Goh criteria [9], which divide patients into those with extensive disease (>20% disease extent at HRCT, or indeterminate disease extent and %pFVC<70%), or limited disease (<20% disease extent at HRCT, or indeterminate disease extent and %pFVC≥70%). Only scans following a HRCT protocol and reconstructed at 1 mm slices were read.

### 2.3 Outcome measures

The primary endpoint was EFS, defined as time in days from development of ILD until death due to any cause, or the development of persistent major organ failure (heart, lung, kidney), defined as left ventricular ejection fraction less than 30% by echocardiography, resting arterial oxygen tension less than 8 kPa (60 mmHg) and/or resting arterial carbon dioxide tension greater than 6.7 kPa (50 mmHg) without oxygen supply, or the need for renal replacement therapy. Secondary endpoints were the change in pulmonary function compared to baseline as a measurement of morbidity, and safety profile defined as treatment-related mortality and number of hospital admissions. Outcome measures of the intensified treatment group were compared with the residual SSc-ILD group treated with what was considered estimated optimal care (EOc) by their treating physician.

### 2.4 Intensified immunosuppressive treatment procedure

The intensified treatment protocol consisted of 7–10 sessions of TPE in a period of 2–3 weeks, 6 months oral cyclophosphamide (2–3 mg/kg, adjusted in case of low leucocyte number) followed by long-term mycophenolate mophetil (MMF). In case of intolerance to maintenance treatment, patients switched to rituximab. In addition prednisone (maximum 30 mg a day, tapering to 5 mg in three to four months) was given. TPE procedures were carried out with Haemonetics MCS+ ©. At each session approximately 50% plasma volume was exchanged, using a 10% albumin solution as replacement fluid.

### 2.5 Statistical analysis

Statistical analysis was performed by using IBM SPSS Statistics for Windows, version 25.0. Descriptive statistics were calculated for demographic and clinical characteristics. Continuous variables are presented as mean (± standard deviation) in case of a normal distribution or median (interquartile range (IQR)) in case of non-normal distribution. Categorical variables are presented as numbers (percentage). Characteristics of each cohort were compared with control group using *t*-test or Mann-Whitney *U* test (whichever appropriate based on variable distribution) for continuous variables and Pearson chi-2 or Fisher exact test for categorical variables.

To calculate SMR, mortality rates of the general population adjusted for age were obtained from the Central Statistical Office of the Netherlands. SMR was defined as the ratio of observed deaths in the SSc cohort to the number of expected deaths of the Dutch age-matched population in 2011–2016. The expected number of deaths is the product of the number of people in each age group of the SSc cohort multiplied by the age-matched mortality rate of the general population. The observed number of deaths was divided by the person time under observation. Cumulative survival rates at 1, 3, 5 and 10 years were computed by the Kaplan-Meier method and significance was tested with the log-rank test. To evaluate whether there was a significant change over time in %pFVC and %pDLco between the two SSc-ILD treatment groups, a linear mixed-effects model with a fixed effect for time (continuous, in years) was used. Figures were made using GraphPad Prism version 5.03 for Windows.

## 3 Results

### 3.1 Survival and standardized mortality rate of the total SSc cohort

**Fig. 2:**
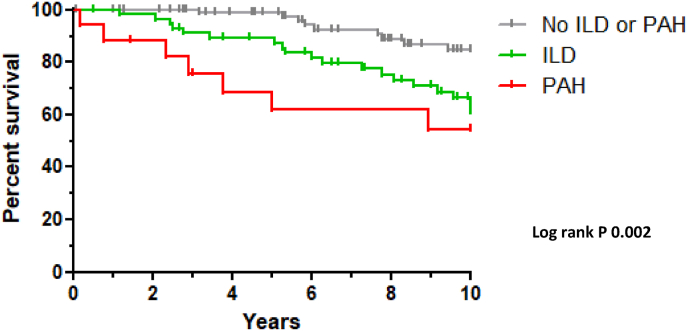
Overall survival SSc cohort from age at disease onset, SSc without ILD and PAH, SSc-ILD and SSc-PAH. Overall survival rates at 1, 3, 5, and 10 years from disease onset in the total SSc cohort were 98.7%, 94.1%, 91.3%, 73.0% and 62.4% (not shown). Overall survival rates at 1, 3, 5 and 10 years from disease onset in the SSc patients without ILD and PAH were 100%, 100%, 98.7% and 84.7%. Overall survival rates at 1, 3, 5 and 10 years from disease onset in the SSc-ILD patients were 100%, 91.0%, 89.2% and 63.7%. Overall survival rates at 1, 3, 5 and 10 years from disease onset in the SSc-PAH patients were 88.2%, 75.6%, 61.9% and 54.1%. Patients without ILD or PAH had significantly better survival rates compared to SSc-ILD (p = 0.005) and SSc-PAH (p < 0.001).

The entire cohort consisted of 161 SSc patients, including 57 SSc-ILD (35.4%) and 17 SSc-PAH (10.6%) patients. Demographics and clinical characteristics of the cohort are shown in Table 1. During the median 8.9 years (IQR 8.6) follow-up there were 48 deaths recorded in the whole cohort of 161 patients, corresponding with an age-adjusted SMR of 3.0 (CI 95% 1.39–5.55). The mean age at death was 71.5 years (±7.6) with a median disease duration of 7.7 years (IQR 8.0). There were 9 deaths (52.9%) reported in the SSc-PAH group, compared to 27 (47.4%) in the SSc-ILD group, corresponding with a collective age-adjusted SMR of 4.1 (CI 95% 1.93–8.60). The age-adjusted SMR in patients without ILD and/or PAH is 1.58 (CI 95% 0.84–4.98). Of 41 patients, the cause of death was identified (Table 2). The number of malignancies of any kind did not differ between patients with (15.6%) or without (6.5%) immunosuppression (p = 0.694). Overall survival rates from disease onset in the total SSc cohort, SSc-ILD and SSc-PAH patients are shown in Fig. 2.

**Table 1:** Demographic and clinical characteristics of the single center Maastricht cohort.

Clinical features	All patients (n = 161)	Without CPI (n = 87)	SSc-ILD (n = 57)	SSc-PAH (n = 17)
**Age at disease onset**^**¥**^**, mean** ± **SD, y**	56.5 ± 14.0	55.1 ± 13.8	56.3 ± 14.0	64.5 ± 12.6
**Female sex**	118 (73.3)	69 (79.3)	32 (56.1)	17 (100)
**Duration of follow-up, median, IQR, y**	8.9 (8.8)	8.4 (9.1)	9.6 (6.6)	5.0 (12.4)
**Skin involvement, n (%) available**	160 (99.4)	87 (100)	56 (98.2)	15 (100)
Limited	114 (70.8)	70 (80.5)	32 (56.1)	12 (70.6)
Diffuse	22 (13.7)	3 (3.4)	18 (31.6)	1 (5.9)
Sine sclerosis	24 (14.9)	14 (16.1)	6 (10.5)	4 (23.5)
**Antibody**				
Centromere	92 (57.1)	69 (79.3)	10 (17.5)	13 (76.5)
Topoisomerase I	36 (22.4)	6 (6.9)	30 (52.6)	0
RNA polymerase III	4 (2.5)	2 (2.3)	2 (3.5)	0
Other	3 (1.8)	1 (1.1)	2 (3.6)	0
ANA only	25 (15.5)	8 (9.2)	13 (22.8)	4 (23.5)
None	1 (0.6)	1 (1.1)	0	0
**Disease manifestations**				
ILD	59 (36.6)	0	57 (100)	2 (11.8)
PAH	33 (20.5)	0	16 (28.0)	17 (100)
Renal crisis	4 (2.5)	0	3 (5.3)	1 (5.9)
GI involvement	101 (62.7)	40 (46.0)	48 (84.2)	13 (76.5)
Digital ulcers	85 (52.8)	44 (50.8)	31 (54.4)	10 (58.8)
**Comorbidities**				
CVD	51 (31.7)	24 (27.6)	19 (33.3)	8 (47.1)
Malignancy	36 (22.4)	17 (19.5)	14 (24.6)	5 (29.4)
**Treatment**				
PAH medication	65 (40.4)	23 (26.4)	26 (45.6)	16 (94.1)
Immunosuppression	104 (64.6)	46 (52.9)	50 (87.7)	8 (47.1)

Data are presented as number (%) unless otherwise indicated. IQR: interquartile range; CPI: cardiopulmonary involvement (ILD or PAH); ILD: interstitial lung disease; PAH: pulmonary arterial hypertension; ANA: anti-nuclear antibodies; GI: gastro-intestinal; CVD: cardiovascular disease (myocardial or cerebral infarction); ¥ Defined as the date of the first non-Raynaud's phenomenon symptom.

**Table 2:** Cause of death in total SSc cohort.

Cause of death n = 48 (29.8%)	
**SSc related n=17 (41.5%)**	
PAH	4
Pulmonary fibrosis	4
Heart disease (arrthymia, heart failure)	5
GI failure (aspiration, ileus)	4
**Not-SSc related n=24 (58.5%)**	
Malignancy	11
Sepsis	9
Ischemic heart or cerebral disease	2
Other	2
**Unknown**	**7**

There were 48 deaths recorded during the median 8.9 years (IQR 8.6) follow-up in the whole cohort of 161 patients. Of 41 patients, the cause of death was identified. Data are presented as numbers. PAH: pulmonary arterial hypertension. GI: gastrointestinal.

### 3.2 Demographics and baseline clinical characteristics in the SSc-ILD cohort

The 57 patients in the SSc-ILD cohort consisted of 32 (56.1%) females and the mean age of ILD onset was 60.5 (±11.9) years. Four patients were lost to follow-up and five patients were excluded from analysis, because of incomplete clinical details. Of the remaining 48 SSc-ILD patients, 24 patients fulfilled the inclusion criteria for the early-onset intensified treatment including TPE. Clinical outcomes were compared with the 24 SSc-ILD patients in the EOc group. Patients in the EOc group were excluded for the early-onset intensified treatment because they were not treatment-naïve (n = 10 earlier immunosuppressive treatment, n = 1 antifibrotic treatment), had a disease duration >3 years (n = 6), suffered from a renal crisis (n = 2), or declined informed consent for TPE (n = 4). Of note, even in the EOc cohort 7/24 patients received TPE, a clinical decision based on inflammatory features such as elevated sIL2r or signs of active alveolitis on the DTPA scan. Demographics and clinical characteristics of the two groups are shown in Table 3. Treatment within the EOc group was heterogeneous, less patients received either cyclophosphamide (50% vs 95.8%; p = 0.001), TPE (29.2% vs 100%; p < 0.001), or maintenance MMF (37.5% vs 83.3%; p = 0.003) (see Table 3).

**Table 3:** Baseline characteristics of the SSc-ILD cohort.

Clinical features	Intensified Treatment (n = 24)	EOc treatment (n = 24)	p
**Age at disease onset**^**¥**^**, mean** ± **SD, y**	57.7 ± 11.7	56.2 ± 14.9	0.247
**Age at onset ILD, mean** ± **SD, y**	58.9 ± 11.9	61.9 ± 12.1	0.639
**Female sex**	11 (45.8)	13 (54.2)	0.564
**Disease duration, median, IQR, y**			
Duration total follow-up	8.8 (5.1)	10.2 (10.6)	<0.001
Duration from first non-RP symptom to ILD diagnosis	0.8 (1.0)	3.3 (9.0)	0.003
Duration follow-up from onset ILD	7.7 (6.4)	5.5 (8.7)	0.048
**Smoking**	10 (41.7)	8 (33.3)	0.627
**BMI median, IQR, kg/m**^**2**^	25.0 (6.8)	23.6 (3.9)	0.061
**Skin involvement**	n = 24 (100)	n = 23 (95.8)	
Limited	10 (41.7)	15 (62.5)	0.106
Diffuse	12 (50.0)	5 (20.8)	0.044
Sine sclerosis	2 (8.3)	3 (12.5)	0.666
**Antibody**			
Centromere	1 (4.2)	7 (29.2)	0.048
Topoisomerase I	17 (70.8)	9 (37.5)	0.020
RNA polymerase III	1 (4.2)	1 (4.2)	1.000
Other	0	1 (4.2)	1.000
ANA only	4 (16.7)	6 (25.0)	0.477
None	1 (4.2)	0	1.000
**Raynaud**	21 (87.5)	24 (100)	0.234
**Pulmonary function tests, mean** ± **SD**	n = 22 (91.6)	n = 21 (87.5)	
TLC % predicted	81.2 ± 17.8	79.0 ± 18.6	0.921
FVC % predicted	84.8 ± 21.5	92.0 ± 25.9	0.449
DLCO/VA % predicted	68.8 ± 16.0	67.1 ± 19.9	0.396
**Ground glass opacification on HRCT thorax**	22 (91.7)	22 (91.7)	
Extensive disease (Goh stage)	11 (45.8)	9 (37.5)	0.537
**DTPA scan**	22 (91.6)	8 (33.3)	
Positive	20 (90.9)	5 (62.5)	0.102
**6MWD**	12 (50.0)	14 (58.3)	
Median, IQR, m	491 (128)	397 (206)	0.548
Distance % predicted	76 (65–94)	63 (28–101)	0.031
**Pulmonary hypertension**	3 (12.5)	4 (16.7)	0.699
**Right heart catherization**	6 (25.0)	5 (20.8)	
mPAP, median, IQR, mmHg	26 (11.5)	35 (21)	0.126
PWP, median, IQR, mmHg	7 (8.8)	10 (7.5)	0.329
PVR, median, IQR, dyn.sec.cm-5	245 (227)	337 (300)	0.556
**Creatinine, median, IQR, umol/L**	77 (19)	83 (30)	0.267
**CRP median, IQR, mg/L**	8.0 (19)	3.0 (11)	0.158
**sIL2r, median, IQR, U/mL**	722 (511)	633 (482)	0.658
**Earlier treatment**	2 (8.3)	10 (41.7)	
Hydroxychloroquine	0	1 (4.2)	1.000
Methotrexate	0	5 (20.8)	0.050
Prednisone	2 (8.3)	8 (33.3)	0.033
**Immunosuppressive treatment first year**			
Plasma exchange	24 (100)	7 (29.2)	<0.001
Cyclophosphamide	23 (95.8)	12 (50.0)	0.001
Ciclosporin + azathioprin	1 (4.2)	1 (4.2)	1.000
Methothrexate	0	3 (12.5)	0.234
Ciclosporin + Rituximab	0	1 (4.2)	1.000
Mycophenolate mofetil	0	1 (4.2)	1.000
No immunosuppression	0	6 (25.0)	0.009
**Immunosuppressive maintenance therapy**			
Mycophenolate mofetil	20 (83.3)	10 (37.5)	0.003
Rituximab	3 (12.5)	3 (12.5)	1.000
Methotrexate	0	2 (8.3)	0.489
Ciclosporin + rituximab	0	1 (4.2)	1.000
Everolimus	0	1 (4.2)	1.000
No immunosuppression	1 (4.2)	7 (29.2)	0.048

Data are presented as number (%) unless otherwise indicated. IQR: interquartile range; BMI: Body Mass Index; TLC: total lung capacity; FVC: forced vital capacity; DLCO/VA: diffusing capacity for carbon monoxide; 6MWD: 6-min walking distance; ANA: antinuclear antibodies; PAP: pulmonary artery pressure; PVR: pulmonary vascular resistance; PWP: pulmonary arterial wedge pressure; RP: Raynaud phenomenon; ¥ Defined as the date of the first non-Raynaud's phenomenon symptom.

Of the 24 included patients in the intensified treatment arm, 21 patients had a disease duration of <1 year. They all received 6 months of oral cyclophosphamide combined with a minimum of 10 cycles of TPE, then followed by maintenance treatment with MMF in 20 patients with a median duration of 60 months (IQR 65). Three patients received rituximab and 1 patient discontinued immunosuppression due to psychosis. One patient initially received combined azathioprine and ciclosporin, but later switched to oral cyclophosphamide combined with TPE. In the intensified treatment group anti-topoisomerase antibodies (70.8% vs 37.5%; p = 0.02) and diffuse skin disease (50.0% vs 20.8%; p = 0.044) were more frequently present. The median duration from first non-RP symptom to ILD diagnosis was shorter in the intensified treatment group compared to the EOc group, with a median of 0.8 years (IQR 1.0) versus 3.3 years (IQR 9.1) (p = 0.003). Baseline pulmonary function testing results, Goh stage, percentage PAH, and inflammatory markers such as sIL2r and CRP were not different between the two groups of SSc-ILD patients. 6MWD as percentage of predicted was lower in the EOc group, but testing results were only available in approximately 50% of patients (Table 3). In 3 patients, Goh stage could not be determined because no HR coupes of the CT were available or alveolar edema was present. In 4 patients no abnormalities were detected on HRCT, the diagnosis of SSc-ILD was then based on abnormal DTPA scan alone.

### 3.3 EFS and cause of death in SSc-ILD cohort

**Fig. 3:**
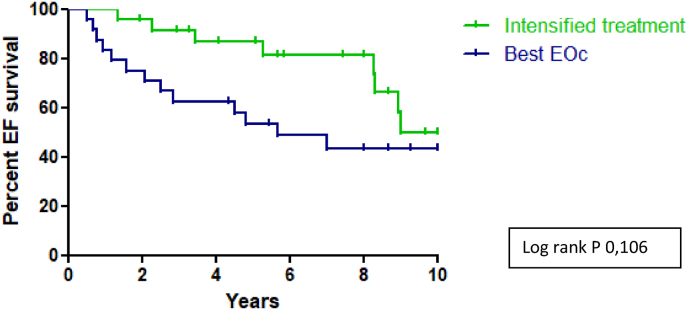
Event-free survival SSc-ILD from onset ILD. EFS rates at 1, 5 and 10 years from onset ILD in the intensified treatment group were 100%, 86.7% and 49.9%. EFS rates at 1, 5 and 10 years were 83.3%, 53.6% and 43.3% in the EOc group (log rank p = 0.106). EFS: event-free survival; ILD: interstital lung disease; EOc: estimated optimal care.

During follow-up from onset ILD 7 deaths (29.2%) were reported in the intensified treatment group and 12 deaths (50.0%) in the EOc group. EFS rates at 1, 5 and 10 years from onset ILD in the intensified treatment group were 100%, 86.7% and 49.9%. EFS rates at 1, 5 and 10 years were 83.3%, 53.6% and 43.3% in the EOc group (log rank p = 0.106; Fig. 3). A SSc-related cause of death occurred in 2 out of 7 patients in the intensified treatment group and in 7 out of 12 patients in the EOc group (not statistically compared due to limited data) (Table 4).

**Table 4:** Cause of death in SSc-ILD patients.

Cause of death	Intensified treatment n = 7 (29.2%)	EOc n = 12 (50%)
**SSc related**		
PAH	0	1
Pulmonary fibrosis	1	0
Heart disease (arrthymia, heart failure)	1	3
GI failure (aspiration, ileus)	0	3
**Not-SSc related**		
Lung cancer	3	3
Respiratory tract infection	2	1
**Unknown**	0	1

Data are presented in numbers. PAH: pulmonary arterial hypertension. GI: gastrointestinal.

### 3.4 Response of interstitial lung disease to treatment

**Fig. 4:**
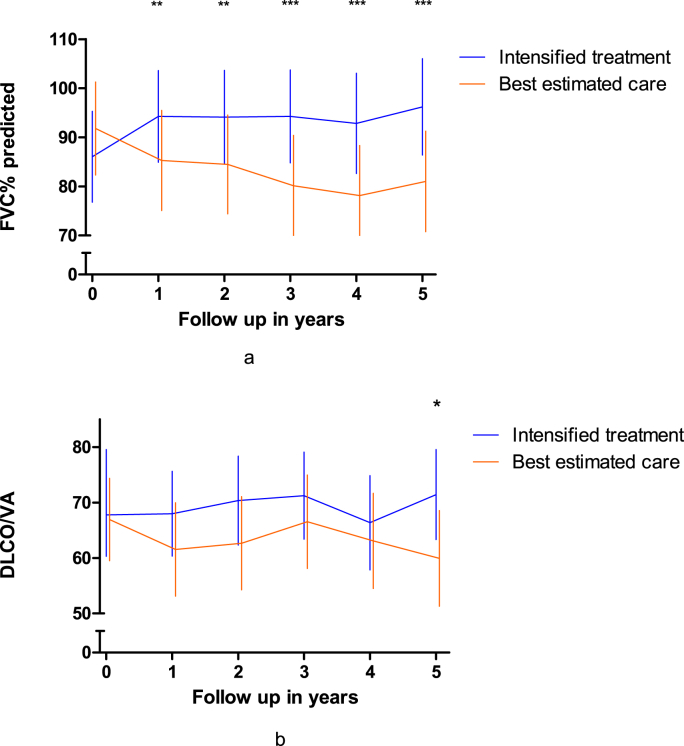
(a). FVC% predicted over time from onset ILD. (b) DLCO % predicted over time from onset ILD.

The change of %pFVC during 5 years in the intensified treatment group showed greater improvement compared to the EOc group (p < 0.001; Fig. 4a). The difference between groups in %pFVC was greatest at 4 years (Δ 20.5%; 95% CI 9.97–31.01). The intensified treatment induced a 10.1% increase of %pFVC as compared to the 10.8% decrease in %pFVC in the EOc arm, over 5 years follow-up. The difference between groups in %pDLco during 5 years showed a Δ of 10.6% (95% CI 1.75–19.57; p = 0,0019; Fig. 4b) in favor of the intensified treatment group, which improved by +3.6%, compared to the *7%* decrease in %pDLco of the EOc group, over 5 years follow-up.

In 20 out of 22 of the patients in the intensified treatment group, DTPA scanning showed active alveolitis. During follow-up 18 out of the 20 positive DTPA scans improved with normalization in 16 cases, 1 patient deteriorated and 1 case remained unchanged. During follow-up no patient in the intensified treatment and 1 patient in the EOc group developed PAH, the latter classified as group 3 PH [8].

### 3.5 Safety of intensified immunosuppressive treatment

Safety of intensified immunosuppression was estimated by evaluating the number of treatment related hospitalizations. In both treatment groups, there were 8 infection-related admissions noted. Other indications were cardiovascular disease (1 vs 2), digital ulcers (11 vs 3) or other (8 vs 10). Treatment-related mortality was low. Only one death during treatment was recorded, in a patient older than 75 years, who died of infection. During follow-up 4 patients in the intensified treatment group developed malignancy and 8 patients in the EOc group (p = 0.182).

## 4 Discussion

In this single-center study investigating a large SSc cohort, we found an age-adjusted SMR of 3.0 which corresponds to reported outcomes in previous studies [10,11]. The age-adjusted SMR of 4.1 in the SSc patients with ILD and/or PAH is even higher, reflecting the high mortality related to cardiopulmonary involvement in these patients. Our treatment study was set up in an exploratory pragmatic way as we felt the urge to treat patients as early as possible with intensified immunosuppression during remission-induction, followed by prolonged maintenance therapy. The rationale for this approach was fueled by the assumption that in the early phase of progressive severe systemic sclerosis immune dysregulation and inflammation is the dominant disease driving factor. At the time we started our study no standardized and well evaluated treatment modalities were available, and in many cases a more restrained approach was usual. To date, HSCT is available, but still only in tertiary centers and selection of patients is still a critical issue.

The 5 years EFS rates in the ASTIS (79.7%) and SCOT (79%) trials (4, 5) is comparable to the 5 years EFS of 86.7% in the intensified and long-term immunosuppressive therapy in our high-risk SSc-ILD patients, but the safety profile in our study appeared to be more favorable, although the direct comparison needs to be interpreted with caution.

Differences in survival can possibly be explained by higher treatment-related mortality, which for instance in the ASTIS trial was 13.9% in the first year of follow up. Treatment-related mortality thus is a scope of interest, and for instance careful screening before selection of patients reduces treatment-related mortality in HSCT [12]. Age at inclusion appeared to matter, as the intensified treatment showed no treatment-related mortality during more than 5 years follow-up in SSc-ILD patients younger than 75 years. Treatment with oral cyclophosphamide during the first 6 months did not lead to more hospitalizations, probably due to clinical experience and close monitoring of leucocyte numbers.

Moreover, pulmonary function in response to intensified treatment improved as compared to the change of pulmonary function in the EOc group. Recovery of pulmonary function, or its residual level after treatment, has great impact on the quality of life. Our study is one of few intervention studies in SSc-ILD that shows both survival and pulmonary function data, reflecting both mortality and morbidity. Randomized controlled trials (RCTs) that compare intensified immune suppression to HSCT in early onset inflammatory progressive SSc are needed. One may also argue whether immunosuppression in the control arm of these well-designed trials has been optimal.

The strength of our study is the inclusion of patients who were at high-risk for disease related mortality and progressive ILD as illustrated by their characteristics: high percentage male sex, anti-topoisomerase antibodies and diffuse skin disease [3,11,[13], [14], [15]]. 12.5% of our patients already had pulmonary hypertension at the start of intensified treatment and 41.7% were current smokers, both associated with worse outcome. We predominantly included patients with early onset SSc-ILD with a median disease duration of 0.8 years from first non-RP symptom, representing the early, immune-inflammatory phase of the disease. The presence of intrapulmonary inflammation was strongly supported by results of initial DTPA scanning, followed by normalization in response to treatment in the majority of cases. The reported outcomes also emphasize the importance of early and aggressive intervention in high-risk patients, even when pulmonary function is still in the normal range to prevent irreversible, fibrotic organ damage [16].

Our study is the first in combining TPE with long-term immunosuppression as treatment for SSc-ILD. Numerous RCTs and clinical studies showed that TPE is a safe and low cost option as additional treatment for SSc, but these studies use different outcome measures and did not use strict regimens of immunosuppression [17]. The mechanism of action remains largely unclear. Persistent injury to endothelial cells, activation of innate and adaptive immunity and downstream effects on smooth muscle cells and fibroblasts results in extracellular matrix production and vascular remodeling [18]. Different auto-antibodies like anti-endothelial cell antibodies (AECAs) may play an important role in fibroblast activation [16,19]. Related to this important role of pathogenic autoantibodies and cytokines in SSc-ILD, TPE seems an effective, steroid-sparing, additional therapy for the induction of clinical remission and subsequent prevention of disease progression [17]. The therapeutic efficacy of the intensified treatment supports the notion that SSc is driven by an aberrant immune response and that the disease process can be reversed [20,21].

The study is limited by its observational design. Another limitation is the small number in both groups, it is however striking that %pFVC and %pDLCO significantly improved in the intensified treatment group. EFS rates in the intensified treatment arm of our study did however not differ from those in the EOc arm, possibly due to the small numbers. The comparison is also hampered by the complexity of the heterogeneous EOc group. One third of these patients were also treated intensively including TPE. Our findings warrant confirmation in larger randomized controlled studies.

In conclusion, SMR in SSc patients with pulmonary involvement is still high. However, early recognition and upfront treatment have great impact on improvement of outcome and prevention of further damage [22]. The DTPA scan can be used as a new and early screening method for the detection of active alveolitis in SSc. Alternative intensified immunosuppressive treatments, with balanced toxicity profiles, are needed [23]. A treatment with TPE and long-term immunosuppressive therapy is safe, has low toxicity, and good overall survival and pulmonary outcome.

## Credit author statement

**J. Potjewijd**: Formal analysis, Investigation, Resources, Writing – original draft, Visualization. **R. Tobal**: Formal analysis, Investigation, Visualization, Writing – review & editing. **D. Silvertand**: Investigation. **H.A. Gietema**: Investigation, Writing – review & editing. **J.G.M.C. Damoiseaux**: Writing – review & editing, Supervision. **P. van Paassen**: Conceptualization, Methodology, Resources, Writing – review & editing, Supervision.

## Declaration of competing interest

The authors declare that they have no known competing financial interests or personal relationships that could have appeared to influence the work reported in this paper.

## Data Availability

Data will be made available on request.
